# The interplay of social group biases in social threat learning

**DOI:** 10.1038/s41598-017-07522-z

**Published:** 2017-08-09

**Authors:** Armita Golkar, Andreas Olsson

**Affiliations:** 1 Karolinska Institutet, Department of Clinical Neuroscience, Division of Psychology, Solna, Sweden; 2 University of Amsterdam, Department of Clinical Psychology, Amsterdam, Netherlands

## Abstract

Learning from other individuals (e.g. social learning) is subjected to biases affecting whom to learn from. Consistent with research in animals, showing similarity-based learning biases and a general tendency to display pro-social responses to in-group individuals, we recently demonstrated that social learning of both fear and safety was enhanced when information was transmitted between same-race individuals. Here, we addressed how two different social group categories jointly affect the transmission of fears by investigating the interplay between racial and supporter group membership. We demonstrate that supporter group membership differentially influenced learning from a racial in-group vs. racial out-group individual. Thus, conditioned skin conductance responses in the same-race condition were significantly higher when fear was transmitted by an in-group (same team) vs. an out-group (rival team) individual, and were related to supporter team identification. However, supporter group membership did not influence learning from a racial out-group demonstrator, suggesting that the presence of an alternative alliance does not necessary reduce the influence of racial biases on social fear learning.

## Introduction

Humans, like other animals, have an evolved capacity to learn by observing the actions and experiences of other individuals, a process referred to as social learning^1^. Social learning may serve adaptive purposes by allowing individuals to acquire knowledge about their environment without the costs associated with first-hand experiences. This advantage is particularly pronounced when learning is associated with risk and potential harm, such as during fear learning^2^. In order to maximize the efficacy of social learning, animals must learn from direct experiences and use social learning strategies selectively depending on the circumstances and the individuals from whom they learn^1, 3, 4^. For example, in the context of learning, cues such as familiarity, relatedness and social status have been shown to modulate the efficacy of fear transmission in non-human animals^5–8^. In humans, transmission of both fear and safety information was recently shown to be enhanced between individuals belonging to the same (in-group) as compared to different (out-group) racial groups^9^, as measured by conditioned autonomic responses. These findings are in line with research suggesting that race acts a default dimension of person perception and categorization^10–12^. It’s not clear, however, whether a racial bias will be under the influence of the socio-cultural context. Although most previous studies have failed to identify conditions that reduce racial categorization^10^, a few notable exceptions have shown that spontaneous and implicit racial categorization are reduced in the context of alternative alliance dimensions^13^, including political context^14^. To date, no study has addressed if coalitional affiliations can reduce racial biases that govern observational fear learning. This is particularly important given that learning from others represents a fundamental route through which humans use past experience to adapt to their environment. Observational fear learning in particular, represents a form of socio-emotional learning that is widespread across taxa, from insects to primates, to learn about threats in their surrounding through social signals and enables learning about dangerous outcomes without incurring the risk of being personally injured. The selectivity of such social learning requires understanding how competing sources of social group categorization bias these learning processes.

Here, we addressed how race and coalitional alliance (soccer supporter membership) of the observed individual jointly affect the transmission of fears measured by conditioned autonomic responses. We used an established observational fear learning paradigm to study the role of group biases in social fear learning^9^, duirng which the participants, acting as observers, are exposed to a video clip depicting a demonstrator. During the *observational acquisition* stage, the observer learned by watching the actions of the demonstrator, who belonged either to their own or a different racial group (racial in/out-group), and who they believed represented a supporter of their own or a rival team (supporter in/out-group). During the video, the demonstrator displayed discomfort when receiving an electrical shock (Unconditioned stimulus, US) paired with the presentation of an image of a snake (reinforced conditioned stimulus, CS+), but never when paired with a spider image (unreinforced CS−). During a subsequent *direct test*, participants were re-exposed to the CSs, without receiving shocks, and in the absence of the demonstrator. To assess learning, we used a standard index of conditioned response (CR) in humans: Skin conductance response (SCR), which reflects the phasic increase in physiological arousal in response to threat.

## Results

**Figure 1:**
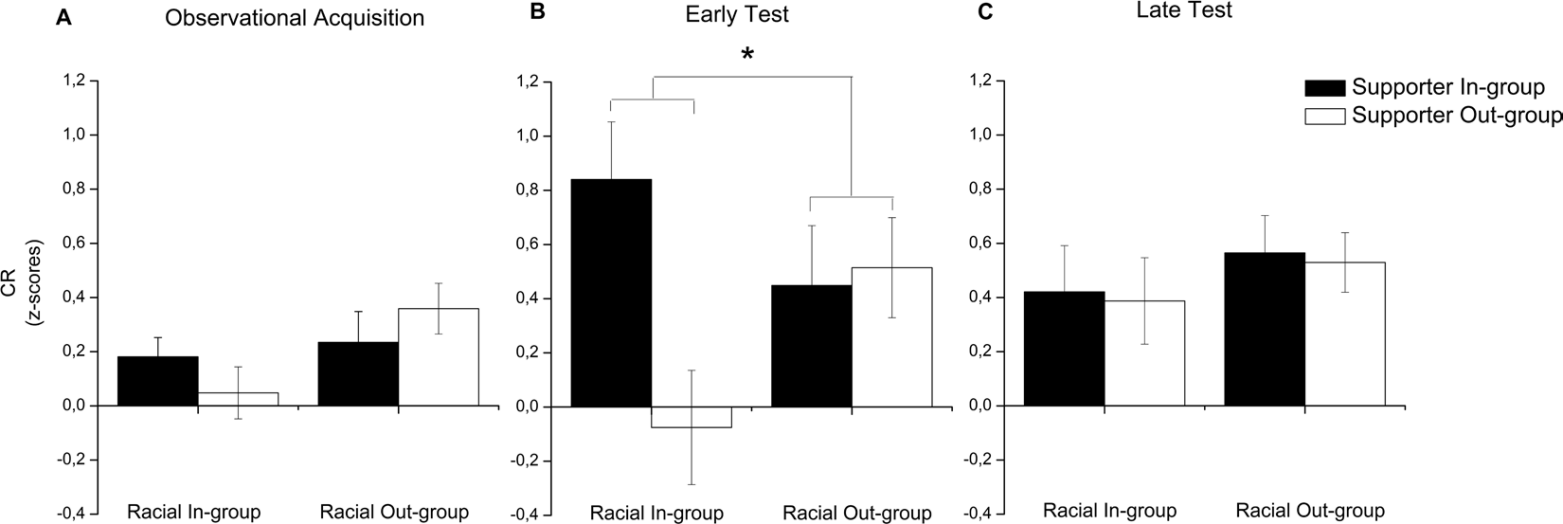
Mean CR (CS+ > CS−) across the experiments displayed for the Racial In-group and Racial Out-group separately as a function of Supporter group membership. (**A**) During observational acquisition SCRs to the CS+ were overall higher than to the CS− in the absence of significant group differences. (**B**) During the early stage of the direct test, supporter group membership significantly modulated CRs but only when fear was transmitted from a racial in-group demonstrator (**C**). During the late stage of the direct test, there were no between group differences left. Errors bars denote standard error of the mean (SEM). *p < 0.05.

The CR was indexed as the differential SCR to the snake (CS+) and spider (CS−) image. The mean CR are presented in Fig. 1. During the observational acquisition stage, fear learning was confirmed by overall higher responses to the CS+ than to the CS− (Main effect of Stimulus: F(1,91) = 18.64, p < 0.001, η_p_^2^ = 0.17). As predicted, CR differed as function of group membership during the critical direct test stage. In line with previous findings^9^, and because CRs extinguish rapidly in the absence of reinforcement (Schiller *et al*., 2010), the effects were most pronounced during the early stage of the direct test (Stimulus × Block × Supporter group × Demonstrator race interaction (F(1,91) = 4.16, p = 0.04, η_p_^2^ = 0.04). Focusing on the early test stage, we first confirmed that CRs differed as a function of Supporter group and Demonstrator race (Stimulus × Supporter group × Demonstrator race interaction (F(1,91) = 5.70, p = 0.02, η_p_^2^ = 0.06). To understand the influence of supporter group membership on the expression of observational fear learning, we analyzed the influence of Supporter group in participants that had observed a racial in- and out-group demonstrator separately. We found that supporter group membership had a significant effect on CRs in the group that learned from a racial in-group demonstrator, Stimulus × Supporter group interaction F(1,45) = 9.50, p = 0.004, η_p_^2^ = 0.17. Thus, there was a difference in CRs when learning from an in-group or out-group supporter, t(45) = 3.08, p = 0.004, 95% CI for the difference between groups = [0.30–1.44], Cohen’s d = 0.90, and follow-up paired samples t-test confirmed that only participants that had observed a demonstrator they believed was a supporter from their own team showed a significant CR (In-group Supporter = t(22) = 3.95, p = 0.001, 95%CI = [0.38–1.23], Out-group supporter: t(23) = 0.35, p = 0.73, 95% CI = [−0.48–0.34]. Supporter group membership did not significantly influence the expression of observationally acquired fear from a racial out-group demonstrator (Stimulus × Supporter group interaction: F(1,46) = 0.05, p = 0.82, η_p_^2^ < 0.001) but CS+ responses were overall higher than CS− responses (Significant main effect of Stimulus F(1,46) = 11.26, p = 0.002, η_p_^2^ = 0.20).

Finally, based on our previous finding that negative attitudes towards the out-group was associated with less expressed learning during the early test stage^9^, we correlated the supporter team identification ratings (as measured by the SSIS) with the CRs during the early stage in the racial in-group condition. We found that higher scores on supporter team identification were associated with less learning from an out-group supporter (r = −0.43, p = 0.04, r^2^ = 0.18.

## Discussion

Here, we investigated how race and a second social group category jointly affect the transmission of fears by crossing Race (Racial in/out-group) with Supporter group (Supporter in/out group). We demonstrate that observational fear learning biases are not limited to racial categories and extend to coalitional affiliations (i.e. supporter group membership), but this influence was significantly more pronounced during racial in-group vs. racial out-group learning. Thus, in the racial in-group condition, supporter group membership enhanced the expression of learning when fear was transmitted from a same-team supporter compared to when fear was transmitted by a rival-team supporter. Moreover, among same-race individuals, supporter team identification scores were negatively related to learning from an out-group supporter, suggesting that social identity might suppress learning from out-group members. However, more research is needed to fully characterize this effect as (1) there is a possibility of a ceiling effect in detecting enhanced in-group learning, and (2) a validated questionnaire measuring rival-group hostility could clarify whether rival-group hostility similarly predicts less learning from an out-group supporter. Supporter group membership did not significantly influence learning when fear was transmitted from a racial out-group demonstrator, suggesting that supporter group identification did not counteract racial categorization. Taken together, this study is the first to demonstrate that supporter group membership bias social fear learning, but that the presence of a supporter alliance did not reduce the influence of race. Rather, supporter group alliance was attended to in the same-race condition without reducing racial categorization.

This pattern of results is predicted by prior research demonstrating that racial categorization is not always reduced when crossed with coalition^15^. Thus, both race and supporter group membership are treated as coalitional cues, but race has a higher prior probability of predicting coalitional affiliation^13^. Therefore, to reduce racial categorization, current models posit that this requires the cues to be presented in a context during which the novel cue (supporter group membership) actively predicts the pattern of alliance, and race does not^14–16^. Thus, given that there is no evidence that race *is not a* predictive cue of affiliation in the current set-up of the stimuli, our findings that supporter group membership bias learning only in the same-race condition are well aligned with current models suggesting step-wise prioritization of coalitional cues in the absence of evidence that race does not predict the ongoing pattern of alliances.

In sum, accumulating evidence from research on social group categorization has established that people spontaneously categorize newly encountered individuals by their sex, age, and race^10–12^. Here, we extend previous research on racial biases in observational fear learning, and demonstrate that supporter group membership similarly biases observational fear learning between same-race individuals, suggesting that it reflects a general system biasing social learning towards individuals belonging to one’s own group. Future research addressing cross-cutting coalitions should investigate how observing interactions between individuals during which a novel cue such as supporter group membership predicts the pattern of alliance, and at the same time race does not actively predict the same pattern of alliance, can reduce racial categorization when learning from other individuals.

## Methods

### Participants

To estimate the required sample size to detect an in-group bias in the expression of observational fear learning, we performed a power calculation (threshold of 80% sensitivity (1− beta = 0.80); significance level 0.05, one-tailed) based on our previous effects of a racial in-group bias using a similar experimental protocol^9^. The power calculation yielded a required sample size below 21 in each group. To achieve this, 95 participants (White Swedish residents of European origin) were recruited from official Supporter webpages and from the Karolinska Institutet campus after filling out the Sport spectator identification scale SSIS^17^, which is a 12-item questionnaire (rated from 1–6) that measures the degree of identification with one’s own team^1^. Two additional participants that scored 3 SD below the mean SSIS scores were incorrectly included and were removed from the sample and not subjected to any further analysis. Data collection was stopped after we had reached the required sample size based on our power calculation and after our recruitment strategy had ceased to recruit any additional volunteers during a 3 month long period. In each experimental condition (Racial in-group/Racial out-group) participants were randomly assigned to two different groups (Supporter in-group/Supporter out-group). Sample size, age and SSIS scores are reported in Table 1. The research was approved by the regional ethical committee at Karolinska Institutet (2102/340-31/4), and was carried out in accordance with the principles of the revised Helsinki Declaration (World Medical Association General Assembly, 2008). Written informed consent was obtained from each participant.

**Table 1:** Sample size, mean age and total scores on the SSIS reported for each experimental group separately. Standard deviations (SD) are given within brackets. SSIS = Sport Spectator Identification Scale.

Supportership	Race	N	Age	SSIS
In-group	In-group	23	28.4 (4.35)	49.04 (4.24)
Out-group	In-group	24	28.6 (7.01)	48.83 (3.90)
In-group	Out-group	24	30.2 (9.01)	49.90 (3.91)
Out-group	Out-group	24	29.8 (7.43)	47.90 (6.26)

### Stimuli

One image of a snake and one image of a spider served as CSs, and four different male individuals (two White and two Black counterbalanced across participants) served as demonstrators (see Supplementary information for details about the stimuli used). Throughout all experimental stages, each CS was presented six times for six seconds in a pseudo-randomized order with an inter-trial-interval (ITI) ranging between 12–18 seconds.

### Experimental procedure

Before the experiment started, participants in both groups were shown a board divided into two sides. On each side of the board, there was a picture of one of two individual demonstrators, each categorized as a supporter of one of the teams. The participants were informed that these two individuals were pre-selected supporters from each team and informed which individual they would observe during the experiment. After this, each participant categorized themselves as belonging to either team by selecting a pin with the team emblem and attaching it to their team’s side. Which individual demonstrator that served as a Supporter in/out-group individual was counterbalanced between participants and team. Importantly, there were no visual cues of supporter membership during the actual experiment that followed, and instructions in the absence of visual cues have been shown to be sufficient for coalition categorization^13^.

All participants first underwent an *observational acquisition* stage. During this stage, participants viewed a video depicting either a racial in-group (White) or racial out-group (Black) demonstrator displaying discomfort when receiving an electrical shock (the US) paired with the presentation of an image of a snake (the CS+), but never when paired with a spider image (the CS−). During a subsequent direct test, participants were re-exposed to the both CSs presented as full-screen images in the absence of the demonstrator. During this stage, participants did not without receive any shocks. Accordingly, CR in the presence of the CS+ was due to social learning taking place during the observational acquisition stage. After the experiments, all participants completed a post-experimental questionnaire, assessing CS-US contingency awareness and rated how they perceived the demonstrator’s reactions to the shock and rated the likeability, attractiveness, and how much they could identify themselves with the demonstrator. To control for snake and spider fear, participants also rated their fears to snakes and spiders. (see supplementary information for details).

### Psychophysiological assessment

SCRs to each CS were measured throughout the experiment and the raw signal was off-line filtered with a low-pass filter (1 Hz) and a high-pass filter (0.05 Hz). For each CS trial, conditioned SCRs were measured as the largest peak-to-peak amplitude difference in skin conductance (in microsiemens, µS) in the 0.5 to 4.5 second window following stimulus onset. Responses below 0.02 µS were scored as zero and data was z-transformed prior to analysis^18^.

### Statistical analysis

Across the experiments, physiological fear responses were based on the mean SCR to the CS+ and CS− for each stage and analyzed using separate mixed analysis of variance (ANOVA) with Stimulus (CS+, CS−) as a within-subject factor and Supporter group (In-group supporter, Out-group supporter) and Demonstrator race (In-group race, Out-group race) as between-subject factor. As CRs extinguish rapidly in the absence of shocks^19^, the *direct test* data in was divided into an early and late stage, defined by the mean first three responses versus the mean last three responses. Based on our previous findings of racial group differences in observational fear learning^9^, we focused our main analysis on the early stage of the direct test. Significance were taken at p < 0.05 and partial η^2^ and Cohens’d are reported as measures of effect size where appropriate. Significant interactions were followed up with separate two-tailed t- tests.

## Electronic supplementary material

Supplementary Information

Dataset1
