# Author Correction: The interplay of social group biases in social threat learning

**DOI:** 10.1038/s41598-018-29056-8

**Published:** 2018-07-18

**Authors:** Armita Golkar, Andreas Olsson

**Affiliations:** 1 Karolinska Institutet, Department of Clinical Neuroscience, Division of Psychology, Solna, Sweden; 2 University of Amsterdam, Department of Clinical Psychology, Amsterdam, Netherlands

Correction to: *Scientific Reports* 10.1038/s41598-017-07522-z, published online 09 August 2017

The Acknowledgements section in this Article is incomplete.

“This research was supported by an Independent Starting Grant (284366; ELSI) from the European Research Council to A. Olsson and a post-doctoral grant from the Swedish Science foundations (2015-00312) to A. Golkar.”

should read:

“This research was supported by an Independent Starting Grant (284366; ELSI) from the European Research Council, and the Knut and Alice Wallenberg Foundation (KAW 2014.0237) to A. Olsson and a post-doctoral grant from the Swedish Science foundations (2015-00312) to A. Golkar.”

