# Supplementary material for: The interplay of social group biases in social threat learning
Source: Sci Rep. 2017 Aug 9;7:7685. doi: 10.1038/s41598-017-07522-z (PMC5550414; doi:10.1038/s41598-017-07522-z)
Supplement: Supplementary file 1 — Supplementary Information [file 41598_2017_7522_MOESM1_ESM.pdf]

The interplay of social group biases in social threat learning

Armita Golkar & Andreas Olsson

Supplementary information

Methods

Stimuli

Conditioned stimuli (CSs). One image of a snake served as the CS+ and one image of a spider served as the CS-. This rationale was based on pilot testing during which assigning the spider or snake image as the CS+ or CS- produced comparable CRs during observational fear learning. To further support this conclusion, we additionally re-analyzed previously published data[1] using the same snake and spider images and temporal parameters as in the current experiment. These analyses revealed that non-reinforced SCR responses to these images did not differ in 2 independent samples (sample 1 (N=41): Main effect of CS: $F(1,39) = .01$ $p = .99$); sample 2 (N=64): Main effect of CS: $F(1,61) = .59$, $p = .45$) when presented alone or when presented in the presence of a learning model. Finally, participants completed two 4-item questionnaires assessing spider and snake fear modified after the Spinnenangstscreening, SAS[2] and we confirmed that there were no significant between-groups differences in snake or spider fear in the current sample (one-way ANOVA: both p's >.9 between groups) and introducing snake and spider fear as covariate did not alter the interpretation of our results (i.e. Stimulus x Supporter group x Demonstrator race interaction remained significant during the test stage, $F(1,85) = 5{,}25$: $p = .024$; $\eta^{2=}$ .06). Spider and snake fear did not significantly interact with any other variable (all p's > .05).

Demonstrators. Participants in both experiments completed a post-experimental interview assessing CS-US contingency awareness (i.e. which CS the demonstrator received a shock to) and rated how they experienced the reactions of the demonstrator when receiving shocks (on a 9-point scale, not at all – very much) by rating a) how much discomfort they thought that the

person in the movie experienced in response to the shocks, b) how expressive he was when receiving the shocks, c) how natural they experienced his reactions to be and d) how much empathy they felt for the person in the movie. Also, participants rated on a 9-point scale (not at all – very much) how much they liked the person in the movie, how attractive the person was and how much they could identify themselves with the person in the movie.

Results

Demonstrators. The questionnaire data was analyzed in multivariate analysis of variance (MANOVA) with significance taken at $p < .005$. We first analyzed between-group differences in how participants rated the reaction of the demonstrator in response to receiving the electrical shocks. This analysis revealed no significant main effect of Supporter group ($F(4,88) = 1.29$, $p = .28$, Wilk's $\Lambda = 0.95$, partial $\eta^2 = .06$), Demonstrator race ($F(4,88) = 1.82$, $p = .13$, Wilk's $\Lambda = 0.92$, partial $\eta^2 = .08$) or Supporter x Demonstrator race interaction ($F(4,88) = 1.66$, $p = .17$, Wilk's $\Lambda = 0.93$, partial $\eta^2 = .07$) on the ratings. Finally, we compared ratings of likeability, identification and attractiveness between groups in a separate MANOVA. This analysis showed that ratings differed significantly based on Supporter group, $F(4,87) = 6.97$, $p < .001$, Wilk's $\Lambda = 0.81$, partial $\eta^2 = .19$. Although ratings were generally more positive for in group demonstrators than for out-group demonstrators, follow-up tests (bonferroni corrected) revealed that only likeability ratings differed significantly between in vs. out-group demonstrators ($F(1,89) = 20,79$, $p <.001$, $\eta^2 = .19$) with higher ratings for in-group demonstrators ($M = 5.46$, $SD = 2.13$) than for out-group demonstrators ($M = 3.55$, $SD = 1.99$).

The interplay of social group biases in social threat learning

Armita Golkar & Andreas Olsson

References

1 Golkar, A., & Olsson, A. 2016. Immunization against social fear learning. *Journal of Experimental Psychology: General* **145**(6). 665.

2 Rinck M., Bundschuh S., Engler S., Muller A., Wissmann J., Ellwart T., Becker E.S. 2002 Reliability and validity of German versions of three instruments measuring fear of spiders. *Diagnostica* **48**(3), 141-149.
